# Robot therapy aids mental health in patients with hematological malignancy during hematopoietic stem cell transplantation in a protective isolation unit

**DOI:** 10.1038/s41598-024-54286-4

**Published:** 2024-02-27

**Authors:** Akiko Yamada, Daigo Akahane, Shiho Takeuchi, Kaori Miyata, Takako Sato, Akihiko Gotoh

**Affiliations:** 1 Department of Hematology, Tokyo Medical University, 6-7-1, Nishi-Shinjuku, Shinjuku-ku, Tokyo 160-0023 Japan; 2 Center for Diversity, Tokyo Medical University, Shinjuku-ku, Tokyo Japan; 3 Department of Nursing, Tokyo Medical University, Shinjuku-ku, Tokyo Japan

**Keywords:** Hematopoietic stem cell transplantation, Chromogranin A, Psychiatry, Quality of life, Robot therapy, Cancer, Health care, Medical research

## Abstract

Patients with hematological malignancy experience physical and psychological pain, such as a sense of isolation and confinement due to intensive chemotherapy in a protective isolation unit (PIU). We examined whether the intervention of a robotic puppy, aibo (manufactured by Sony), could improve patients' mental health as an alternative therapy for pet therapy, which is not feasible in PIU. This study included 21 patients undergoing allogeneic hematopoietic stem cell transplantation (HSCT) (n = 16) or autologous HSCT (n = 5). The patients were randomly divided into the aibo and control groups. Psychological effects were regularly assessed by measuring the levels of salivary stress hormone chromogranin A (CgA), serum oxytocin, and serum cortisol and the quick Inventory of Depressive Symptomatology Self-Report (QIDS-SR) scores. The aibo group demonstrated a significant decrease in CgA level, while the control group showed the opposite trend. In addition, changes in serum oxytocin and cortisol levels indicated that aibo helped reduce stress. There was no significant difference in the QIDS-SR scores between the two groups; however, the psychomotor activity in the aibo group improved significantly. These findings suggest that aibo intervention during a stay in a PIU can improve the mental health of patients with hematological malignancies who have undergone HSCT.

## Introduction

In recent years, research on long-term psychological processes and social support for patients with cancer as a chronic disease has progressed^1^. Furthermore, observational studies on the occurrence of psychological factors in patients with hematological diseases have been performed^2^. Unfortunately, patients with hematological diseases experience physical pain and other complications such as infections, fever, nausea, vomiting, diarrhea, mucositis, nephrotoxicity, pulmonary toxicity, and neurotoxicity due to adverse events associated with intensive chemotherapy^3^. In addition, severe side effects during stem cell transplantation (SCT) due to chemotherapy-related toxicities, engraftment syndrome, and acute graft-versus-host disease have been reported^3^.

Further, the patients experience psychological pain, such as a sense of isolation and confinement in a protective isolation unit (PIU) during hematopoietic stem cell transplantation (HSCT). Patients in a PIU are more likely to experience insomnia and depression than those in standard wards^4^. Several studies have found that animal therapy is effective in the mental health care of pediatric patients and patients with chronic diseases^5,6^. Consequently, dog therapy is becoming more common in hospitals. However, because of the immunocompromised state of patients in a PIU, dog therapy is not feasible due to the potential spread of infections. Notably, the findings of a study comparing the therapeutic effects of dog therapy and a pet-type robot on healthy children suggested that a pet-type robot was a suitable alternative^7^.

This study aimed to examine whether a pet-type robot, aibo (manufactured by Sony), could help improve the mental health of adult patients with hematological diseases undergoing HSCT in a PIU.

## Results

### Patient backgrounds

Twenty-three patients were included in the study; however, two dropped out due to clinical reasons, such as difficulty in collecting samples, leaving the analysis with 21 patients. Table 1 shows the patient characteristics. Eleven patients were included in the aibo group and 10 in the control group. No significant differences in age, sex, diagnosis, disease status, conditioning regimen of SCT, or duration of PIU stay were observed between the two groups.

**Table 1:** Baseline patient characteristics.

SCT	SCT-A	SCT-C	*p*-value
Number of patients	11	10	
Age median (range)	48	57	N.S
	(24–65)	(44–67)	
Sex			
Male	4	7	N.S
Female	7	3	
Diagnosis			
AL	6	4	N.S
MDS	4	2	
Others^#^	1	4	
Disease status at administration in PIU			
CR1-2	5	7	N.S
NonCR	6	3	
SCT			
RIST	10	6	N.S
ASCT	1	4	
Disease status at discharge from PIU			
CR	10	10	N.S
NonCR	1	0	
PIU duration (days) median(range)	61	46	N.S
	(21–109)	(24–88)	

^#^Others included NHL and MM. AL: acute leukemia, ASCT: autologous stem cell transplant, CBT: umbilical cord blood transplant, CR: complete response, Chemo-A: chemotherapy with aibo, Chemo-C: chemotherapy as control, MDS: myelodysplastic syndrome, MM: multiple myeloma, NHL: non Hodgkin lymphoma, PIU: protective isolation unit. RIST: reduced-intensity conditioning stem cell transplant, SCT-A: stem cell transplant with aibo, SCT-C: stem cell transplant as control, SCT: stem cell transplant.

A comparison of the blood sampling parameters for each group at the time of admission is shown in Supplemental Table S2. No significant differences were found in most parameters; however, a significant difference was detected in the total protein level. Although the reason for this result remains to be determined, there were no differences in albumin and fasting blood glucose levels, which are indicators of nutritional status, and the results may reflect differences in serum globulin levels due to inflammation or immune response. The possibility that this influenced the results of this study does not seem likely.

### Salivary chromogranin A (CgA) concentration

**Figure 1:**
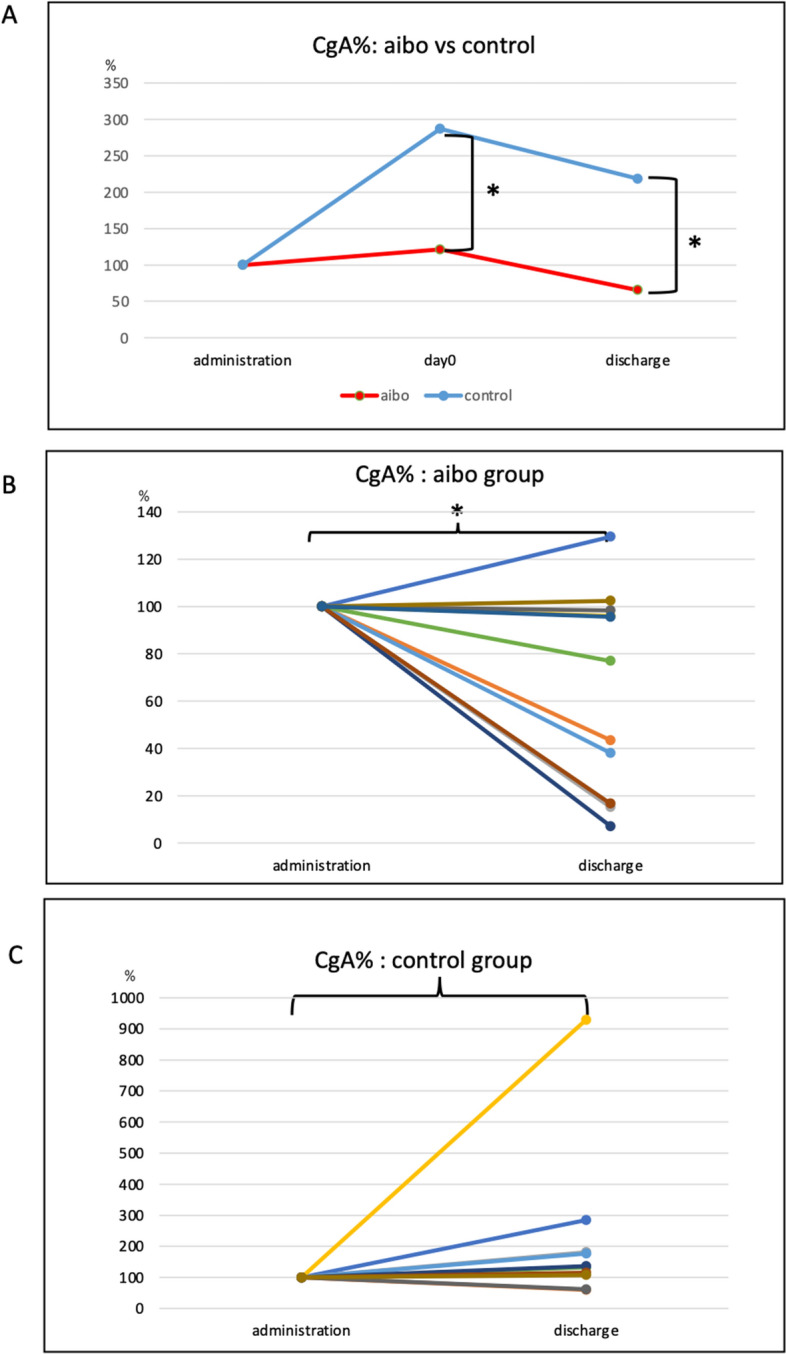
(**A**) Percentage changes in salivary CgA levels at admission in a PIU, on the day of stem cell transplantation (day 0), and upon discharge from a PIU in the aibo and control groups. The aibo group exhibited a percentage change of 121 ± 135% on day 0, while the control group showed a percentage change of 287 ± 358% (*p* = 0.043, Mann–Whitney test). At discharge, the aibo group demonstrated a percentage change of 65 ± 42%, whereas the control group showed a percentage change of 218 ± 258% (*p* = 0.003). (**B**) Levels of salivary CgA in the aibo group at admission in a PIU and discharge from a PIU (0.89 ± 0.79 pmol/mL protein vs. 0.39 ± 0.30 pmol/mL protein, *p* = 0.004). (**C**) Levels of salivary CgA in the control group at admission in a PIU and discharge from a PIU (0.484 ± 0.390 pmol/mL protein vs. 0.826 ± 0.740 pmol/mL protein, *p* = 0.085). ]: Mann–Whitney test. }: paired *t*-test. *Significantly different (*p* < 0.05). CgA: Chromogranin A, PIU: protective isolation unit.

Supplemental Figure S1A, B shows the salivary CgA concentration of each patient at admission to a PIU and from the day of SCT (day 0) to the day of discharge. The Mann–Whitney test was used to compare the salivary CgA levels of the aibo and control groups at admission in a PIU (0.89 ± 0.79 pmol/mL protein vs. 0.484 ± 0.390 pmol/mL protein, p = 0.132), on the day of SCT (day 0; 1.03 ± 0.97 pmol/mL protein vs. 0.74 ± 0.60 pmol/mL protein, p = 0.863), and upon discharge from a PIU (0.389 ± 0.310 pmol/ml protein vs. 0.826 ± 0.740 pmol/mL protein, *p* = 0.152). We examined the percentage change between day 0 and day of discharge using the concentration at admission as the standard (100%) because baseline concentrations varied among patients. Figure 1A shows the percentage change in CgA for both the aibo and control groups, with a peak at day 0 and a decrease at discharge. At discharge, the average decrease in CgA, which indicates mental stress, was greater in the aibo group than that in the control group. Changes in CgA concentration for each patient revealed a significant decrease in the aibo group at discharge compared to that at admission (Fig. 1B). In the control group, there was no significant change or increase in CgA levels (Fig. 1C).

To verify that salivary CgA concentration was not correlated with physical stress, the relationship between salivary CgA concentration and Karnofsy Performance Scale (KPS) score and that between oral intake and Numerical Rating Scale (NRS) score were investigated as an assessment for physical functioning and stress.

### Serum oxytocin concentration

Serum oxytocin concentration levels of each patient at PIU admission and from day 0 to day of discharge from a PIU are shown in Supplemental Fig. S2A, B.

**Figure 2:**
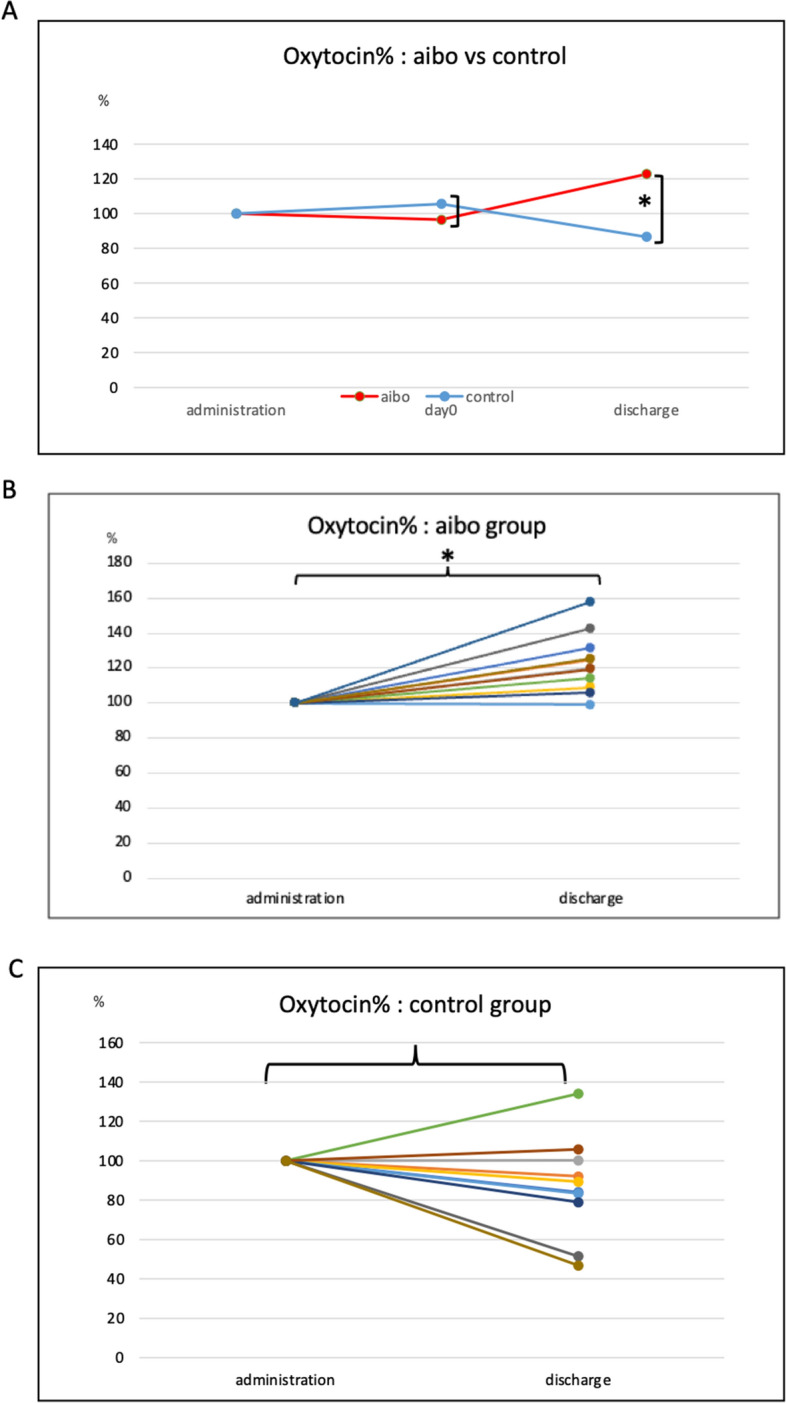
(**A**) Percentage changes in serum oxytocin levels at admission in a PIU, on the day of stem cell transplantation (day 0), and upon discharge from a PIU in the aibo and control groups. The percentage changes on day 0 were 95 ± 11% in the aibo group and 105 ± 31% in the control group (*p* = 0.349). At discharge, the aibo group showed a percentage change of 122 ± 16%, whereas the control group showed a percentage change of 86 ± 25% (*p* = 0.001). (**B**) Serum oxytocin levels in the aibo group at admission in a PIU and discharge from a PIU (388.3 ± 78.0 pg/mL vs. 468.2 ± 72.0 pg/mL, *p* < 0.001). (**C**) Serum oxytocin levels in the control group at admission in a PIU and discharge from a PIU (402.4 ± 107.3 pg/mL vs. 344.3 ± 112.6 pg/mL, *p* = 0.079). ]: Mann–Whitney test. }: paired *t*-test. *Significantly different (*p* < 0.05). CgA: Chromogranin A, PIU: protective isolation unit.

The Mann–Whitney test was used to compare serum oxytocin levels of the aibo and control groups at admission to a PIU (388.3 ± 78.0 pg/mL vs. 402.4 ± 107.3 pg/mL, *p* = 0.654), on the day of SCT (day 0; 403.0 ± 72.7 pg/mL vs. 390.1 ± 73.8 pg/mL, *p* = 0.512), and upon discharge from a PIU (468.2 ± 72.0 pg/mL vs. 344.3 ± 112.6 pg/mL, *p* = 0.008). Thus, Fig. 2A shows that the percentage changes in oxytocin levels for both the aibo and control groups with no significant change noted from PIU admission to day 0. However, the secretion increased significantly in the aibo group at discharge. At discharge, the increase in oxytocin level, which indicates decreasing mental stress, was more pronounced in the aibo group. Furthermore, when the levels at PIU admission and discharge from a PIU for each patient were compared, the aibo group demonstrated a significant increase in oxytocin secretion (Fig. 2B). In contrast, the control group showed a significant decrease in oxytocin secretion (Fig. 2C).

### Serum cortisol concentration

Serum cortisol concentration levels of each patient at PIU admission, day 0, and discharge from a PIU are shown in Supplemental Figure S3A-B. The Mann–Whitney test was used to compare serum cortisol levels between the aibo and control groups at admission in a PIU (880.9 ± 517.5 pg/mL vs. 715.96 ± 667.3 pg/mL, *p* = 0.512), on the day of SCT (day 0; 623.2 ± 595.9 pg/mL vs. 795.4 ± 1397.1 pg/mL, *p* = 0.654), and upon discharge from a PIU (370.3 ± 485.3 pg/mL vs. 1143.5 ± 1260 pg/mL, *p* = 0.043). Thus, a significant decrease in serum cortisol level was observed at discharge.

**Figure 3:**
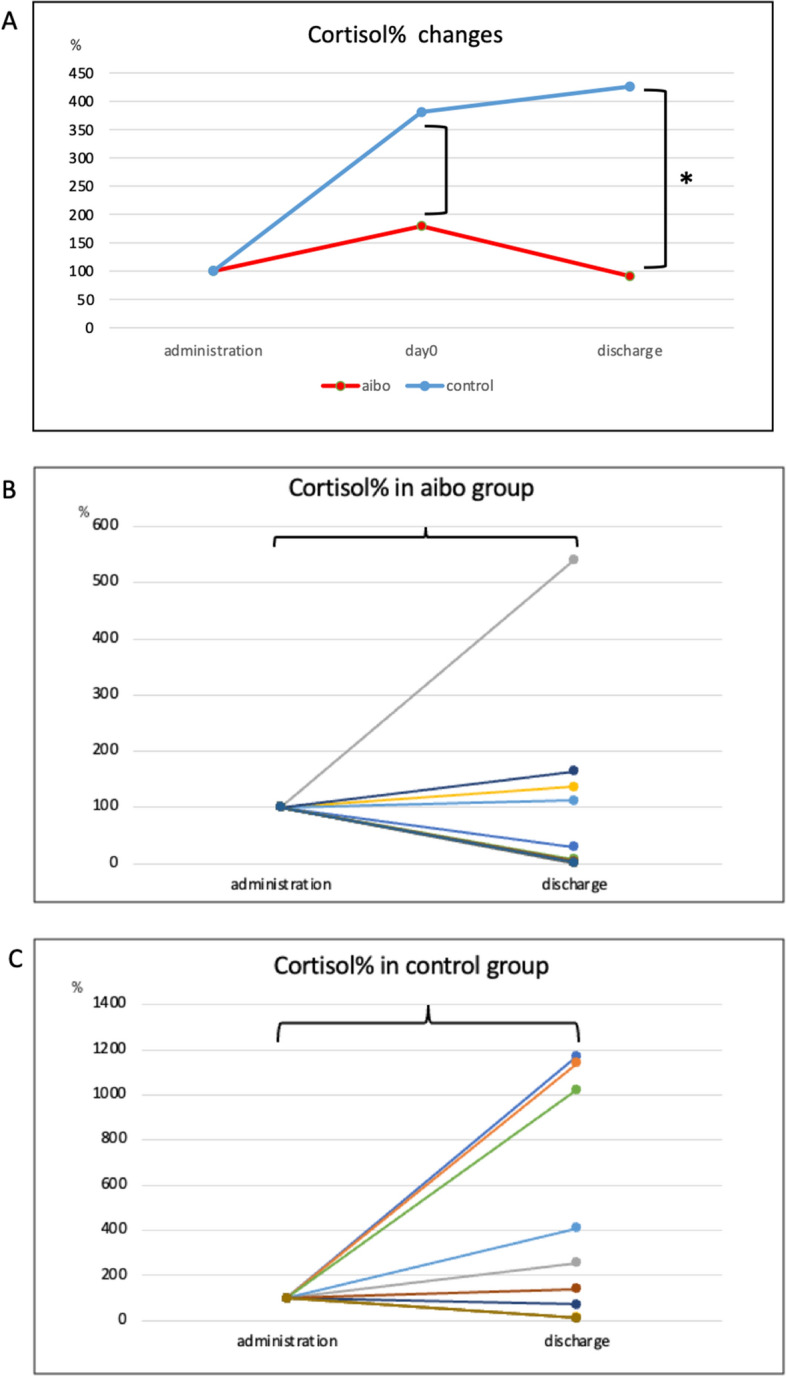
(**A**) Percentage changes in serum cortisol levels at admission in a PIU, on the day of stem cell transplantation (day 0), and discharge from a PIU in the aibo and control groups. The percentage changes on day 0 were 179 ± 301% in the aibo group and 380 ± 757% in the control group (*p* = 0.918). At discharge, the aibo group exhibited a percentage change of 91 ± 161%, whereas the control group showed a percentage change of 425 ± 491% (*p* = 0.024). (**B**) Levels of serum cortisol in the aibo group at admission in a PIU and discharge from a PIU (880.9 ± 517.5 pg/mL vs. 370.3 ± 485.3 pg/mL, *p* < 0.07). (**C**) Levels of serum cortisol in the control group at admission in a PIU and discharge from a PIU (715.96 ± 667.3 pg/mL vs. 1143.5 ± 1260 pg/mL, p = 0.389). ]: Mann–Whitney test. }: paired *t*-test. *Significantly different (*p* < 0.05). CgA: Chromogranin A, PIU: protective isolation unit.

Figure 3A shows that the percentage changes in cortisol levels for the aibo and control groups with no significant change noted from admission to day 0; however, the secretion increased in the aibo group at discharge. At discharge, the decrease in cortisol level, which indicates a decrease in mental stress, was more pronounced in the aibo group than that in the control group.

The t-tests revealed no discernible trend for increasing or decreasing cortisol levels in each group (Fig. 3B, C).

### Assessment using quick inventory of depressive symptomatology-Japan (QIDS-J)

**Figure 4:**
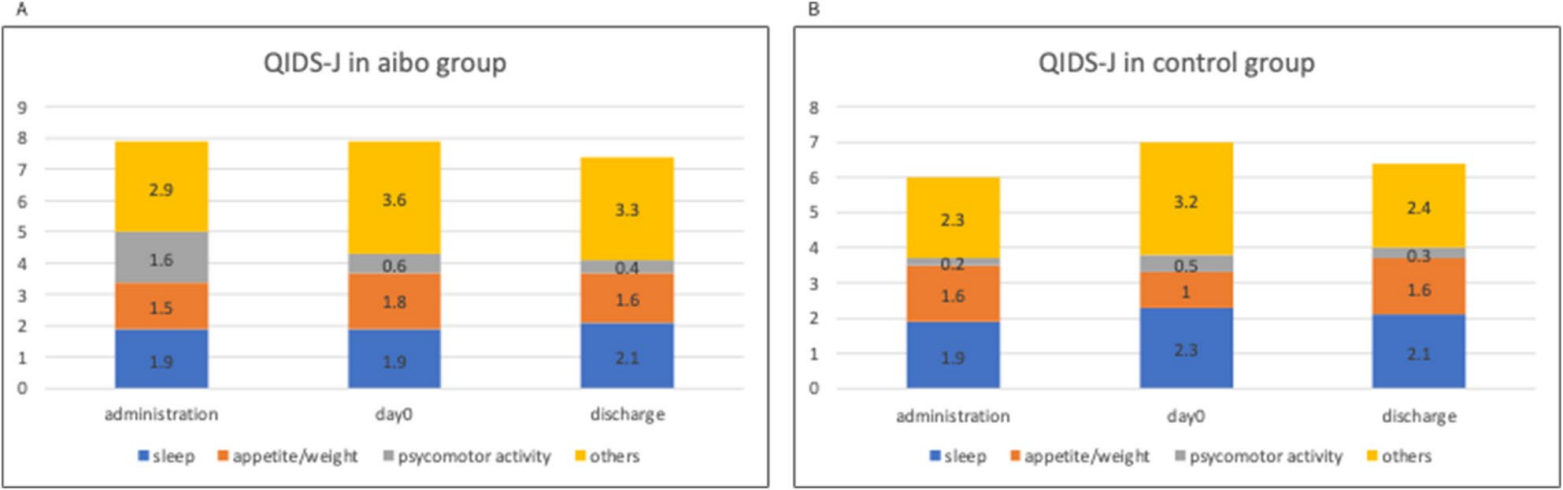
Mean levels of QIDS-J across 3-time points in the aibo group and control group. (**A**) Mean QIDS-J scores in the aibo group at admission in a PIU, on the day of stem cell transplantation (day 0), and upon discharge from a PIU. (**B**) Mean QIDS-J scores in the control group at admission in a PIU, on day 0, and upon discharge from a PIU. QIDS-J: Quick Inventory of Depressive Symptomatology-Japan, PIU: protective isolation unit.

The QIDS-J was used for evaluation of the total scores and sleep, appetite/weight, psychomotor activity, and other variables (Fig. 4A, B). No statistically significant differences were observed in the mean scores between the groups with and without aibo.

Correlations between the QIDS-J and KPS scores and between oral intake and NRS scores were also examined. In addition, correlations with other measures used in this study, such as CgA level, were also examined. The QIDS-J scores were significantly related to KPS scores and oral intake (Supplementary Table S3).

### Assessment of KPS, oral food intake, and NRS

**Figure 5:**
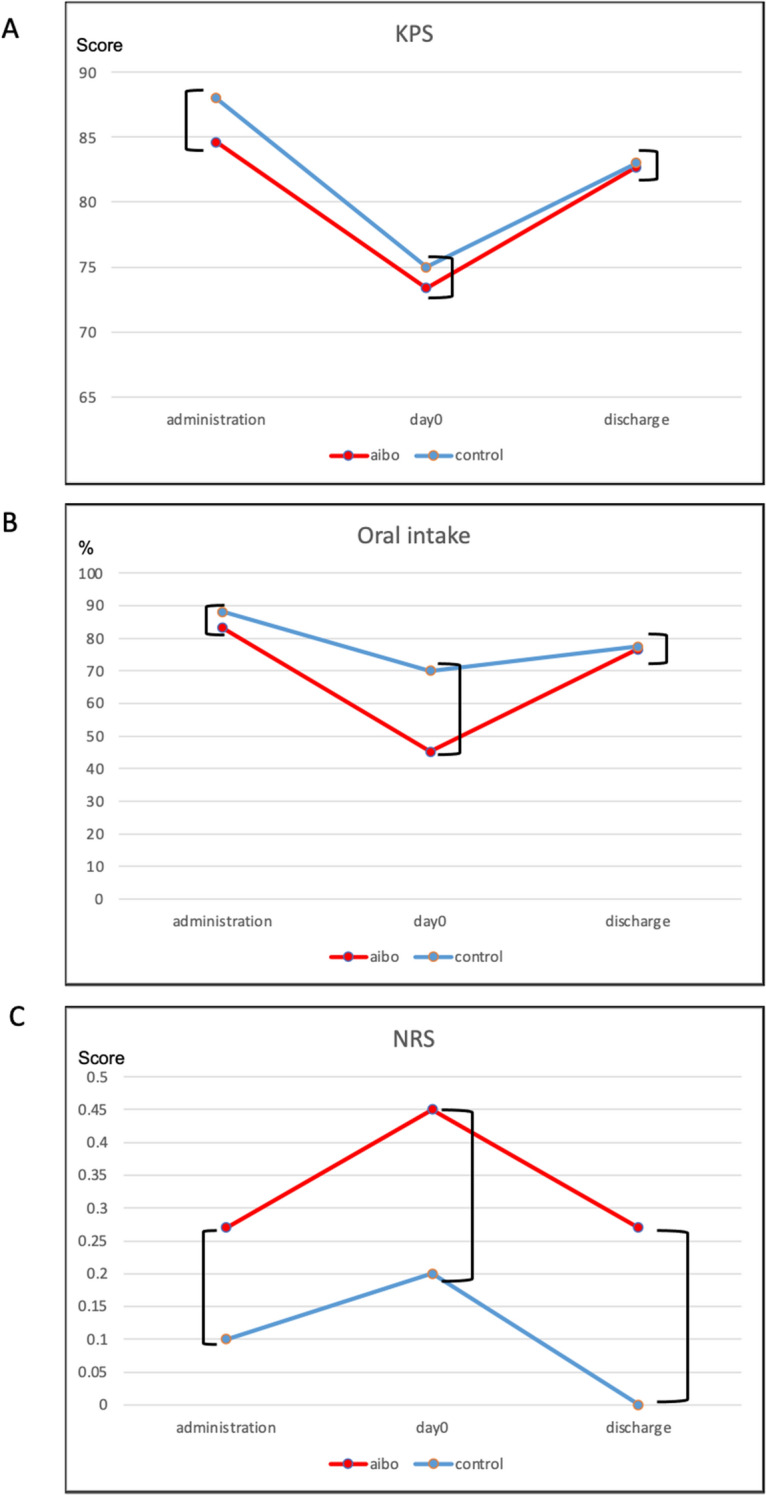
(**A**) Comparison between KPS scores at admission in a PIU, on the day of stem cell transplantation (day 0), and upon discharge from a PIU in the aibo and control groups. KPS scores were the following: at admission to PIU (84.6 ± 10.4 vs. 88.0 ± 4.2, *p* = 0.387), on day 0 (73.6 ± 9.2 vs. 75.0 ± 8.5, *p* = 0.605), and upon discharge from PIU (82.7 ± 11.9 vs. 83.0 ± 8.2, *p* = 0.918) in the aibo and control groups. (**B**) Comparison between oral intake at admission in a PIU, on day 0, and upon discharge from a PIU in the aibo and control groups. %Oral intake was the following: at admission to a PIU (83.2 ± 22.5% vs. 87.7 ± 22.3%, *p* = 0.557), on day 0 (45.1 ± 39.4% vs. 70.1 ± 38.3%, *p* = 0.223), and upon discharge from a PIU (76.6 ± 21.6% vs. 77.4 ± 20.1%, *p* = 0.863) in the aibo and control groups. (**C**) Comparison between NRS scores at admission in a PIU, on day 0, and upon discharge from PIU in the aibo and control groups. NRS scores were the following: at admission to a PIU (0.27 ± 0.47 vs. 0.10 ± 0.32, *p* = 0.512), on day 0 (0.45 ± 0.69 vs. 0.20 ± 0.63, *p* = 0.387), and upon discharge from PIU (0.27 ± 0.647 vs. 0.0 ± 0.0, *p* = 0.512) in the aibo and control groups. ]: Mann–Whitney test. KPS: Karnofsy Performance Scale, NRS: Numerical Rating Scale, PIU: protective isolation unit.

The relationships between salivary CgA concentration or QIDS-J and KPS scores and that between oral intake and NRS scores were investigated to assess physical functioning and stress (Supplemental Figure S3). The trends and averages of these parameters of each patient at admission to a PIU, day 0, and discharge from a PIU were also compared and are shown in Fig. 5.

The Mann–Whitney test was used to compare the KPS scores between the aibo and control groups at admission to PIU (84.6 ± 10.4 vs. 88.0 ± 4.2, *p* = 0.387), on the day of SCT (day 0; 73.6 ± 9.2 vs. 75.0 ± 8.5, *p* = 0.605), and upon discharge from a PIU (82.7 ± 11.9 vs. 83.0 ± 8.2, *p* = 0.918). Oral intake was shown in percentage based on each patient's sex and body type, calculated using the percentage of the oral daily caloric intake. The Mann–Whitney test was used to compare oral intake between the aibo and control groups at admission to a PIU (83.2 ± 22.5% vs. 87.7 ± 22.3%, *p* = 0.557), on the day of SCT (day 0; 45.1 ± 39.4% vs. 70.1 ± 38.3%, *p* = 0.223), and upon discharge from a PIU (76.6 ± 21.6% vs. 77.4 ± 20.1%, *p* = 0.863). The Mann–Whitney test was also used to compare the NRS scores between the aibo and control groups at admission to a PIU (0.27 ± 0.47 vs. 0.10 ± 0.32, *p* = 0.512), on the day of SCT (day 0; 0.45 ± 0.69 vs. 0.20 ± 0.63, *p* = 0.387), and upon discharge from a PIU (0.27 ± 0.647 vs. 0.0 ± 0.0, *p* = 0.512). No significant differences were found in each group.

## Discussion and conclusions

### CgA

In this study, the salivary CgA concentration in the aibo group was significantly lower than that in the control group at discharge, indicating that the aibo intervention improved the mental health of patients admitted to a PIU.

A method for measuring human CgA using specific antibodies was developed in 1998^8^. Several reports have been published on stress assessment using salivary CgA, a useful marker for mental stress because its level increases during mental stress, decreases after withdrawal from stressful situations, and is unresponsive to physical stress^9,10^. Previous studies have shown little variation with sex or age, and the resting salivary CgA concentration in healthy adults ranges from 0.5 to 2.5 pmol/mg protein^9,10^. Further, salivary CgA concentration has also been reported to peak upon waking up, then rapidly decrease and reach its lowest level in approximately 1 h, before stabilizing at a low level throughout the day^8,11^.

Salivary CgA levels were used as an indicator of mental stress. Physiological stress is measured using various parameters, such as blood test findings, saliva, urine, electrocardiogram parameters, blood pressure, and heart rate. We chose salivary substances as a noninvasive indicator instead of such vital signs, which can easily fluctuate depending on physical conditions. In particular, salivary substances, cortisol, and catecholamines are commonly used to assess stress^12,13^. However, these substances have also been found to reflect physical stress^14,15^. Further, a study compared salivary CgA, cortisol, and catecholamine levels and found that cortisol and catecholamine levels increased in acute and chronic pain; nevertheless, salivary CgA level responded only to chronic pain^15^. Notably, pain can be a physical and psychological stressor, whereas acute pain can be life-threatening, unlike chronic pain, and higher physical stress levels are thought to prevent an increase in salivary CgA concentration^16^. Salivary CgA levels have also been found to increase in response to mental stress loads, such as speech and pre-test^17^, but not in response to physical loads, such as ergometers^18^. In addition, in previous studies, salivary CgA concentrations have been used as an indicator of the stress reduction response in situations involving psychological stress^19,20^. The findings of our study reveal that the use of a pet-type robot can help reduce mental stress in patients who underwent HSCT and were transferred to a PIU.

### Oxytocin

The present study raises several concerns regarding the evaluation of oxytocin levels. Firstly, the lack of standardization in assessing sex-specific differences in oxytocin secretion and reference ranges across studies precludes meaningful comparisons of findings. Secondly, the patients enrolled in the study may have experienced hypogonadism due to the intense chemotherapy and total body irradiation, which included the central nervous system, during preconditioning^21^. Consequently, their oxytocin secretion may have been impaired when compared to that of individuals with normal oxytocin levels. Thirdly, it is worth noting that the aibo group had a higher proportion of women, albeit with no statistically significant difference. Notably, oxytocin is often referred to as a "female hormone."

Oxytocin is an attachment-forming hormone, but the mechanism of its secretion remains largely unknown. Estrogen, a female hormone, stimulates oxytocin production and secretion, whereas testosterone, a male hormone, inhibits oxytocin secretion; moreover, it is thought that the sex of a patient may influence its secretion^22^.

Oxytocin levels fluctuate with the menstrual cycle while decreasing with menopause, anxiety, depression, and insomnia^23^. It should be noted that some of the female patients in this study were in a pseudo-menopausal state due to intense chemotherapy; nevertheless, statistically significant differences were not observed in the comparison of serum oxytocin levels between men and women (Supplementary Figure S4). Therefore, sex differences need to be investigated in future studies. This however does not negate the finding that oxytocin levels increased in the aibo group, and the stress reduction effect expected from aibo cannot be denied.

### Cortisol

The levels of cortisol were measured because it is a common stress hormone; however, the distribution of the concentrations varied greatly from case to case, as did the kinetics (Fig. 4B, C). Cortisol concentration may have been affected because all the patients in this study received steroid treatment for graft-versus-host disease following HSCT. Furthermore, serum cortisol levels may have been affected by treatment, especially in patients with lymphoid malignancies, which often includes steroids, leading to transplantation therapy^24,25^. Despite the potential limitation, the cortisol concentration at discharge was significantly higher in the control group than that in the aibo group (Fig. 4A). Furthermore, the cortisol concentration results indicated lower stress levels at discharge in the aibo group.

Notably, CgA is a hormone secreted by the adrenal glands; however, secondary adrenal insufficiency is typically an adrenocortical insufficiency that is rarely accompanied by adrenal medullary insufficiency and is unlikely to be affected by steroid administration. No other cross-sectional study has investigated the multiple endocrine functions of patients throughout the transplant treatment period. Thus, CgA may be more reliable than cortisol for assessing mental stress in patients with hematological malignancies.

### QIDS-J

No significant difference was observed in the total QIDS-J score; however, our findings indicated that psychomotor activity improved in the aibo group (aibo group: 1.6 → 0.5 vs. control group: 0.2 → 0.3, *p* > 0.05). Furthermore, the QIDS-J scores, KPS scores, and oral intake were found to be correlated. The QIDS-J incorporates activities and appetites that are reasonably related. Furthermore, correlations between the QIDS-J and KPS scores and between oral intake and NRS scores were examined, as well as those with CgA. The QIDS-J scores were significantly associated with the KPS score and oral intake. Therefore, the QIDS-J is a physical symptom index that may be inappropriate for assessing mental stress alone. The measurement of anxiety using questionnaires did not show any significant change in a previous study that showed decreased salivary CgA concentration with anxiety reduction^26,27^. These findings suggest that salivary CgA concentrations could be used to assess anxiety, depression, and stress, even when subjective methods are ineffective.

### The usefulness of a pet-type robot for human wellbeing

Along with the physical and psychological side effects of cancer and chemotherapy, patients with cancer also face economic and social impacts. Previous research has shown a tendency for patients with cancer to be more concerned about the economic burden of cancer treatment than the associated physical and psychological side effects. Furthermore, social isolation due to illness is linked to cancer prognosis^28^. Such concerns significantly impact patients, even if the treatment is progressing. Moreover, while most cancer survivors adapt well to life after cancer, some experience lingering negative emotions, such as cancer-related fears, post-traumatic stress, anxiety, and depression. Although mood fluctuations may not meet the criteria for clinical diagnosis, subtle symptoms can hinder the quality of life. Research suggests that behavioral interventions, such as cognitive-behavioral therapy and pharmacological treatment, can effectively address these distressing emotions^29^. In the future, the contribution of robots to such behavioral interventions is expected.

Case studies have investigated how older people with dementia who regularly interact with pet robots (e.g., aibo and paro) experience improved quality of life and reduced loneliness^26,27^. We found that aibo could improve mental stress in adult patients with hematological diseases in a PIU. Robot therapy is effective for patients with dementia and for adults without dementia. We believe that every robot has the potential to help treat stress; however, the advantage of aibo over other pet-type robots is that the exterior of the aibo is not covered with hair, making it easier to prevent contact infection via the robot in immunocompromised patients. During the current coronavirus disease 2019 (COVID-19) pandemic, robots are expected to be especially useful. In this study, hand sanitization before and after contact with aibo and regular cleaning of aibo surfaces had no negative consequences, such as increased infectious diseases in the aibo intervention group (data not shown). Being “fluffy,” as in paro and LOVOT, is an emphasized point with regard to helping in recovery; nevertheless, from the standpoint of hygiene, it may be safer to be "slippery”. However, concerns regarding the effects of social and cultural backgrounds, such as upbringing history, remain. Although the population is somewhat homogenized in Japan, similar efforts in other countries with different cultures may yield different results, which must be considered in future studies.

In addition, the current COVID-19 pandemic had not yet occurred when the research concept was initiated. Previously, family visits to PIUs were permitted under certain conditions. However, visits were strictly prohibited due to the pandemic, leading to more loneliness and stress, increasing the likelihood that a pet robot will be used more effectively.

We had good communication between ward staff and patients owing to aibo. In some cases, aibo sang, danced, and encouraged patients to exercise alongside, which led to a moderate exercise habit, even while the patient was in bed. Therefore, aibo may contribute to the maintenance and improvement of activity of daily living and mental health. In the interviews, many patients reported positive relaxation effects in the aibo intervention group.

In recent years, medical practitioners have been required to provide not only medical treatment but also comprehensive care, including that related to psychosocial factors, to patients. Along with patients, medical practitioners also face psychosocial challenges^30^. Robot intervention can improve the quality of communication between patients and medical practitioners and reduce the burden on medical practitioners in providing psychosocial care. Indeed, a previous study has shown that social robots affect mental health care^31^.

Although the usefulness of social robots in medicine has been demonstrated in some of these aspects, their effectiveness in reducing medical costs and hospital admissions warrants further investigation.

Finally, there is an important concern in this study. Even in this group of patients, physical pain from the treatment was severe from days 0 to 21, and we observed that the time spent with aibo during this period was shorter (data not shown). Further, these findings indicated that aibo was incapable of mitigating physical burdens and stress from day 0 to 7 during HSCT. Thus, we may be able to optimize the impact of aibo only after the phase of physical recuperation. Consequently, determining the suitable timing for maximizing effectiveness may be imperative.

## Material and methods

### Patients

This study included Japanese patients hospitalized at the Tokyo Medical University Hospital who underwent SCT and were treated in a PIU between February 2020 and August 2022. The Medical Ethics Review Board of the Tokyo Medical University (T2019-0172) approved this study. All patients involved in the study provided written informed consent for the utilization of their medical data in accordance with the declaration of Helsinki.

### Pet-type robot “aibo”

**Figure 6:**
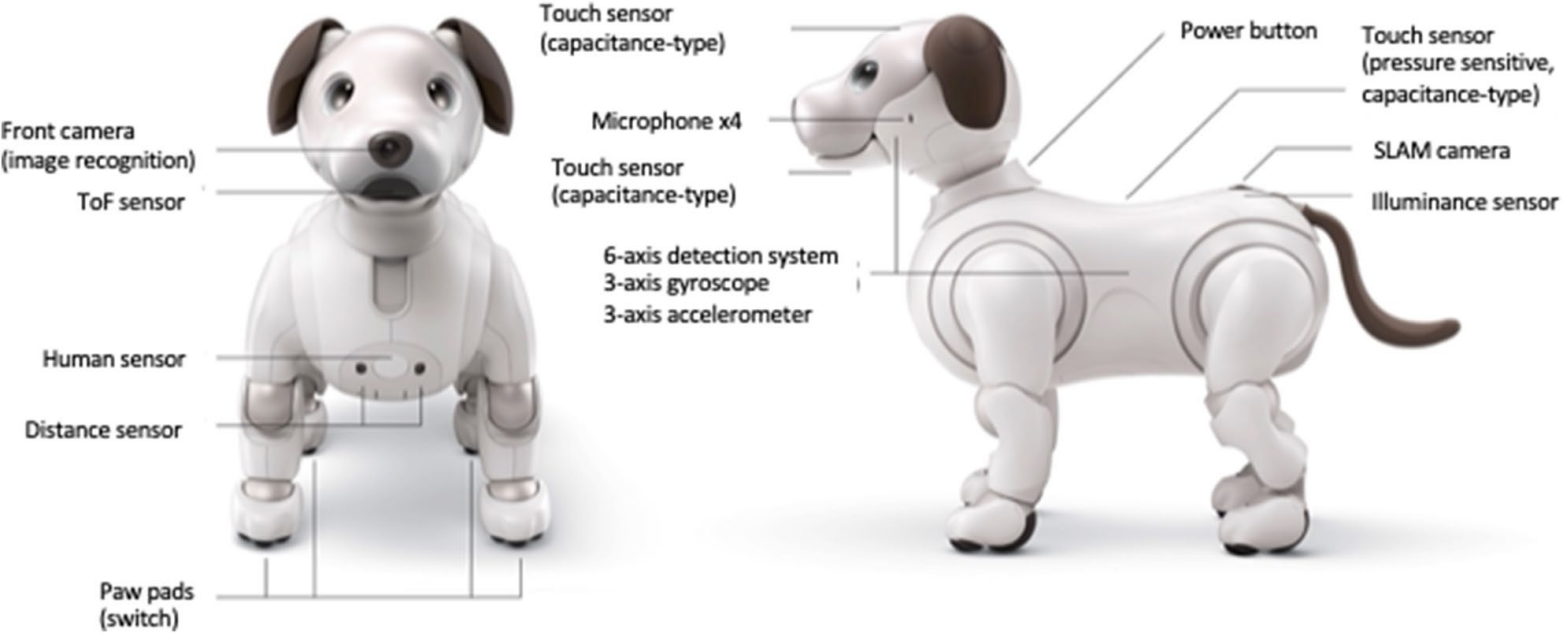
The study utilized “aibo,” a robotic device manufactured by Sony. The accompanying image was provided by Sony.

We used aibo (ERS-1000), an autonomous entertainment robotic puppy manufactured by Sony (Sony Corporation, Tokyo, Japan) (Fig. 6). Aibo recognizes the user and the surrounding environment using sensing technology; subsequently, it uses this information to understand the situation at a high level and decide what approach to take, including how to think about the situation and what action to take using artificial intelligence and deep learning. The personality of aibo is formed by mapping the surrounding environment and people who interact with it, remembering experiences of being praised and discouraged, and taking on the role of a unique animal.

### Design

#### Data collection contents and methods

Patients were randomly divided in the order of enrollment of eligible patients into the aibo and control groups. At the time of admission, patients with cognitive impairment or poor performance status (PS > 2) were excluded, along with those with mental disorders, such as schizophrenia or bipolar disorder, or those deemed inappropriate for the study by a physician. Each patient in the aibo group was allowed to “pet” an aibo in a PIU during the treatment period, and each patient was free to name the aibo. Psychological effects were regularly recorded weekly from the time of admission to discharge from a PIU. The patients were assessed by measuring three types of stress hormones: (1) salivary CgA, (2) serum oxytocin, and (3) serum cortisol and the QIDS-J—a depression screening questionnaire.

The data supporting this study’s findings are not openly available. However, they are available from the corresponding author upon reasonable request in a controlled access repository where necessary.

#### Measurement of salivary chromogranin A

Salivary CgA is a hormone that has recently gained prominence as a stress biomarker as it indicates the activation of the hypothalamic-sympathetic-adrenal system and is released from the adrenal medulla in the biological response to mental stress^11^. Salivary CgA concentrations have previously been reported to range between 0.5 and 2.5 pmol/mg protein in healthy patients^9^.

To avoid contamination by oral microparticles, saliva samples were collected using the straw method from spontaneous drooling 1 h of oral care after lunch in this study^32^. The samples were immediately stored at − 30 ºC, and their concentrations were determined using an enzyme-linked immunosorbent assay with a YK070 Human Chromogranin ELIZA Kit (Yanaihara Research Institute, Japan). All samples were assayed in duplicates. Salivary CgA measurements were expressed per total salivary protein concentration using the Lowry method to correct for the salivary secretion effect.

#### Measurement of serum oxytocin

Oxytocin is a neuropeptide secreted from the posterior pituitary gland that has recently been revealed to play a role in reproductive physiology and social cognition, anxiety, maternal behavior, and various other behaviors^33,34^. Furthermore, previous studies revealed increased oxytocin levels in participants after interacting with or petting animals^35^.

Leftover blood samples, which were collected before breakfast, from routine blood tests were frozen at –30ºC until laboratory analysis. The DetectX Oxytocin ELISA Kit (Arbor Assays LLC, USA) was used to perform an enzyme-linked immunosorbent assay to determine oxytocin concentration. All samples were assayed in duplicates.

#### Measurement of serum cortisol

The main glucocorticoid produced and secreted by the adrenal cortex is cortisol, which is known as the “stress hormone” because it is involved in stress response and influences the effects of stress adaptation, including blood pressure and blood glucose levels. Previous studies have revealed that isolation has a negative impact on mental health, increasing cortisol levels^36^. Further, serum cortisol levels are associated with the severity of depression and suicide risk^37^. Additionally, service dogs for children with autism have resulted in reduced cortisol levels^38^. In this study, the DetectX Cortisol ELISA Kit (Arbor Assays LLC, USA) was used to perform an enzyme-linked immunosorbent assay to determine cortisol concentration using the frozen blood samples. All samples were assayed in duplicates.

#### QIDS-J

The Quick Inventory of Depressive Symptomatology Self-Report (QIDS-SR) has been reported to be useful in determining depression levels in patients^39^. All patients in this study were Japanese; hence, QIDS-J^40^, the Japanese version of the QIDS-SR, was used. The QIDS-SR is used to measure the frequency and severity of depressive symptoms in the previous week, using 16 parameters, including sleep, appetite, weight, psychomotor activity, and others. The score is reported on a 27-point scale where 0–5 is normal, 6–10 is mild, 11–15 is moderate, 16–20 is severe, and 21–27 is very severe, with higher scores indicating more depressive symptoms; Supplemental Table S1 lists the questionnaire. The cut-off point between remission and non-remission of depression is between 6 and 7 points, which is considered optimal based on its sensitivity and specificity. In addition to assessing the severity of depression, the QIDS-SR is a diagnostic tool for major depressive disorders, as defined by the Diagnostic and Statistical Manual of Mental Disorders-IV diagnostic criteria of the American Psychiatric Association. In the United States, it has been used as a tool to assess depression symptoms over time in large-scale clinical trials, such as the Sequenced Treatment Alternatives to Relieve Depression^41^. The QIDS-J was regularly scored and recorded as a subjective assessment of stress status in this study.

### Statistical analysis

Statistical analyses were performed using IBM SPSS Statistics version 26 (IBM, Armonk, NY, USA). Values are expressed as mean (± standard error). Student’s t-test, chi-square test, and Fisher’s exact test were used to compare patient backgrounds, depending on the data characteristics. To compare stress hormone concentration, QIDS-J scores, and blood sampling parameters, paired samples *t*-test and Mann–Whitney test were performed. Spearman’s rank correlation coefficient was used to examine the correlation between stress hormone concentration, QIDS-J score, and other physical indices, such as the KPS score, oral food intake, and NRS score. Statistical significance was defined as *P* < 0.05.

### Supplementary Information

Supplementary Information 1.

Supplementary Information 2.

Supplementary Information 3.

Supplementary Information 4.

Supplementary Information 5.

Supplementary Information 6.

Supplementary Information 7.

## Data Availability

The data supporting the findings of this study are available from the corresponding author, AY, upon reasonable request.
