# Supplementary material for: Robot therapy aids mental health in patients with hematological malignancy during hematopoietic stem cell transplantation in a protective isolation unit
Source: Sci Rep. 2024 Feb 27;14:4737. doi: 10.1038/s41598-024-54286-4 (PMC10899246; doi:10.1038/s41598-024-54286-4)
Supplement: Supplementary file 1 — Supplementary Information 1. [file 41598_2024_54286_MOESM1_ESM.docx]

**Supplemental Table 1** Quick Inventory of Depressive Symptomatology (QIDS SR)

QIDS-SR includes Sleep disturbance (initial, middle, and late insomnia or hypersomnia) (Q 1 - 4), Sad mood (Q 5), Decrease/increase in appetite/weight (Q 6 - 9), Concentration (Q 10), Self-criticism (Q 11), Suicidal ideation (Q 12), Interest (Q 13), Energy/fatigue (Q 14), Psychomotor agitation/retardation (Q 15 - 16).

To scoring, each score are added: the highest score on any 1 of the 4 sleep items (Q1-4), the highest score on any 1 of the appetite/weight items (Q6-9), the highest score on either of the 2 psychomotor items (Q15 and 16), sum total of the other items(Q5,10-14). Severity of depression can be judged based on the total score. 1-5 = No depression, 6-10 = Mild depression, 11-15 = Moderate depression, 16-20 = Severe depression, 21-27 = Very severe depression.
