# Supplementary material for: Robot therapy aids mental health in patients with hematological malignancy during hematopoietic stem cell transplantation in a protective isolation unit
Source: Sci Rep. 2024 Feb 27;14:4737. doi: 10.1038/s41598-024-54286-4 (PMC10899246; doi:10.1038/s41598-024-54286-4)
Supplement: Supplementary file 2 — Supplementary Information 2. [file 41598_2024_54286_MOESM2_ESM.docx]

|  | **SCT-A** | **SCT-C** | **p-value** |
| --- | --- | --- | --- |
| **WBC (/μL)**  **(range)** | 375.5 | 305 | N.S. |
|  | (130-1290) | (100-440) |  |
| **Hb (g/dL)**  **(range)** | 9.527 | 10.23 | N.S. |
|  | (7.1-12) | 4(.7-15.9) |  |
| **PLT(×10*4/μL)**  **(range)** | 16.56 | 16.27 | N.S. |
|  | (2.3-30.4) | (4.2-29.7) |  |
| **TP (g/dL)**  **(range)** | 6.41 | 5.89 | 0.029* |
|  | (5.5-7.7) | (5.4-6.4) |  |
| **Alb (g/dL)**  **(range)** | 4.1 | 3.88 | N.S. |
|  | (3.7-5) | (3.6-4.2) |  |
| **eGFR (mL/min/1.73㎡)**  **(range)** | 102.87 | 85.4 | N.S. |
|  | (72.7-133.9) | (59-123.9) |  |
| **FBG (mg/dL)**  **(range)** | 92.36 | 99.1 | N.S. |
|  | (80-112) | (76-150) |  |
| **CRP (mg/dL)**  **(range)** | 0.8091 | 0.6 | N.S. |
|  | (0.02-2.97) | (0.02-0.17) |  |

**Supplemental Table 2 Laboratory Data at administration in PIU**

The average laboratory results of blood samples at the time of administration in a PIU are shown.

FBG: fasting blood glucose, PIU: protective isolation unit, SCT-A: stem cell transplant with aibo, SCT-C: stem cell transplant as control, SCT: stem cell transplant.
