# Supplementary material for: Robot therapy aids mental health in patients with hematological malignancy during hematopoietic stem cell transplantation in a protective isolation unit
Source: Sci Rep. 2024 Feb 27;14:4737. doi: 10.1038/s41598-024-54286-4 (PMC10899246; doi:10.1038/s41598-024-54286-4)
Supplement: Supplementary file 3 — Supplementary Information 3. [file 41598_2024_54286_MOESM3_ESM.docx]

|  | vs.CgA | | vs. QIDS-J | |
| --- | --- | --- | --- | --- |
| Physical assessment | r_s_ | p-value | r_s_ | p-value |
| KPS | -0.023 | 0.774 | -0.244 | 0.002** |
| Oral intake | 0.055 | 0.349 | -0.342 | 0.001** |
| NRS | -0.015 | 0.81 | 0.109 | 0.174 |
| QIDS-J | -0.061 | 0.454 | - | - |

**Supplemental Table 3** Correlation between CgA or QIDS-J and KPS, oral intake and NRS

There was no clear correlation between CgA and physical assessments. On the other hand, QIDS-J showed a weak correlation with KPS and oral intake.

CgA: Chromogranin A, KPS: Karnofsky Performance Scale, NRS: numerical rating scale, r_s_: spearman’s rho correlations.
