# Supplementary figures and images for: Robot therapy aids mental health in patients with hematological malignancy during hematopoietic stem cell transplantation in a protective isolation unit
Source: Sci Rep. 2024 Feb 27;14:4737. doi: 10.1038/s41598-024-54286-4 (PMC10899246; doi:10.1038/s41598-024-54286-4)

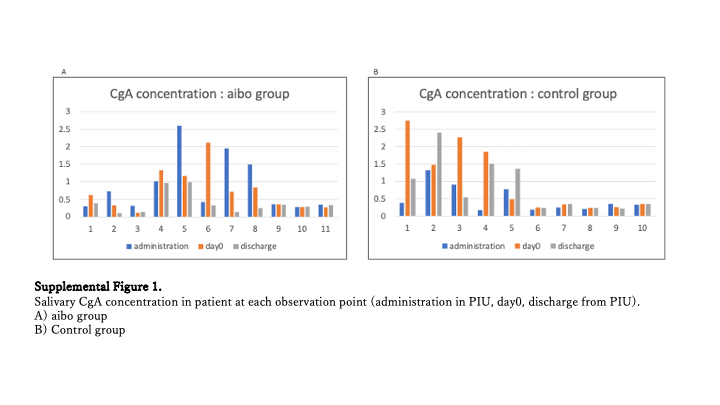

Supplement: Supplementary file 4 — Supplementary Information 4. [file 41598_2024_54286_MOESM4_ESM.tiff]

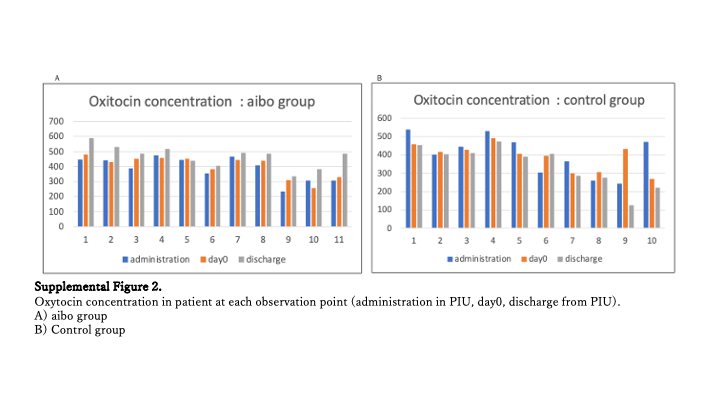

Supplement: Supplementary file 5 — Supplementary Information 5. [file 41598_2024_54286_MOESM5_ESM.tiff]

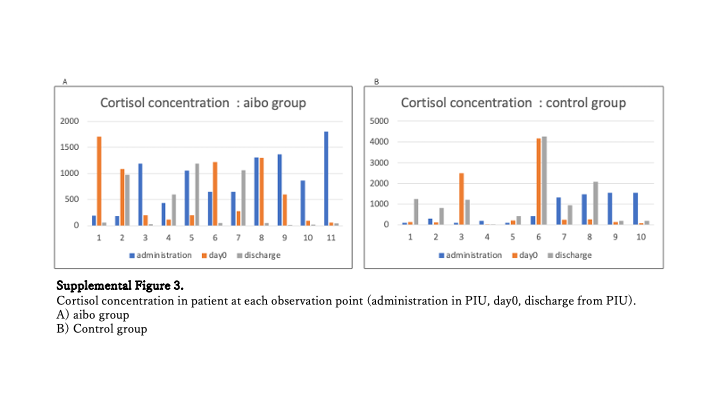

Supplement: Supplementary file 6 — Supplementary Information 6. [file 41598_2024_54286_MOESM6_ESM.tiff]

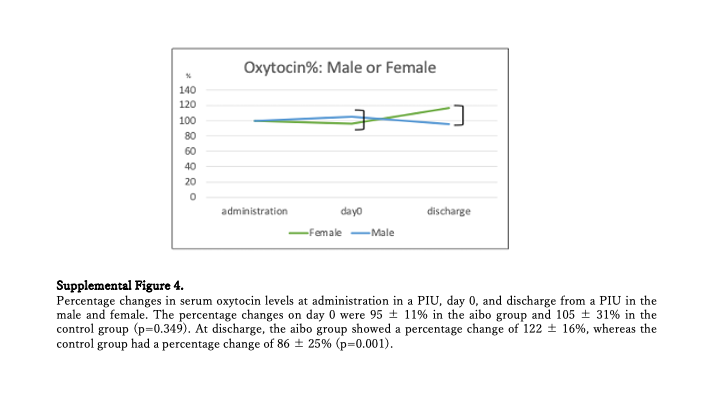

Supplement: Supplementary file 7 — Supplementary Information 7. [file 41598_2024_54286_MOESM7_ESM.tiff]
